# Cultural Management of Terror and Worry During the COVID-19 Pandemic: How Religiosity and a Dream of Human Solidarity Help the Polish People Cope

**DOI:** 10.3389/fpsyg.2021.790333

**Published:** 2021-12-10

**Authors:** Arkadiusz Gut, Łukasz Miciuk, Oleg Gorbaniuk, Przemysław Gut, Anna Karczmarczyk

**Affiliations:** ^1^Department of Cognitive Science, Faculty of Philosophy and Social Sciences, Nicolaus Copernicus University in Toruń, Toruń, Poland; ^2^Faculty of Philosophy and Social Sciences, Institute of Psychology, Nicolaus Copernicus University in Toruń, Toruń, Poland; ^3^Institute of Psychology, The John Paul II Catholic University of Lublin, Lublin, Poland; ^4^Faculty of Philosophy, The John Paul II Catholic University of Lublin, Lublin, Poland; ^5^Department of Cognitive Science, Nicolaus Copernicus University in Toruń, Toruń, Poland

**Keywords:** COVID-19, religiosity, solidarity, terror management theory, cultural worldview, Polish

## Abstract

The COVID-19 pandemic, which involves the threat of contracting a potentially fatal disease, can be understood as a source of terror. According to terror management theory, people shield themselves from terror by adopting culturally specific worldviews and protecting self-esteem. The study investigates the protective role of worldviews that are culturally specific to Poles: religiosity and social solidarity. The hypothesis was that Poles who tend to worry, entertain these worldviews and are more likely to maintain high self-esteem and concentrate on the current moment (carpe diem), which theoretically allows them to reduce future-related anxiety. Path analysis confirmed that self-esteem, the centrality of religiosity, and expectation of solidarity due to the COVID-19 pandemic mediate the relationship between the worry trait and carpe diem.

## Introduction

### The Coronavirus Disease 2019 Pandemic as a Source of Terror

The outbreak of the coronavirus disease 2019 (COVID-19) pandemic has reminded people around the world of the fleeting nature of life. Suddenly people were forced to face a threat of getting a contagious and potentially fatal disease, losing their loved ones, and suffering from severe economic consequences of the pandemic. At the same time, they may have experienced a sense of loneliness, helplessness, and desperation, emerging from the exposure to an ongoing stressful situation that they could not escape. Because the world has changed dramatically and unexpectedly, personal constructs people formerly used in their attempts to understand the world and cope with life turned out to be useless or at least insufficient. Crucial personal constructs that call for a quick redefinition due to the COVID-19 pandemic include work, education, interpersonal contacts, safety, healthcare, individual and social responsibility. According to the classical view, situations in which personal constructs cease to fulfill their functions and a person feels unable to control potential threatening events are likely to produce anxiety (Kelly, 1955; Bandura, 1986). As an unavoidable, long-term situation saturated with experiences of unsafety and unpredictability related to a permanent threat to life and health, the COVID-19 pandemic is “loaded with mortality salience” (Steele, 2020, p. 98) and produces a significant amount of terror (Solomon et al., 1991; Pyszczynski et al., 2020; Rossi et al., 2020).

### Coping With Terror in the Light of Terror Management Theory

Terror Management Theory (TMT) addresses the pervasive role that death awareness plays in human life and focuses on endeavors people undertake to cope with death-related existential terror (Pyszczynski et al., 2015). It is essential that the sense of mortality motivates humans to seek means that will help them symbolically transcendent their own existence (Greenberg et al., 1986). In order to protect themselves from the permanent feeling of anxiety and to function effectively in everyday life, people need to view their life as meaningful in the face of death. According to the TMT, people provide themselves with a sense of security and meaning in life by developing a particular worldview and then living in line with it (Greenberg et al., 1986; Solomon et al., 1991; Hayes et al., 2008). This may increase self-esteem, reduce anxiety, and in sum, foster good adaptation, especially in times when the inevitability and unpredictability of death are immanent (Pyszczynski et al., 1999; Heidegger, 2010; Bulut, 2020). In line with TMT, to mitigate the potential terror provoked by the awareness of death, people very often recourse to religious worldviews. It is emphasized that religious beliefs are particularly well suited to shield against the terror associated with increased death awareness (Vail et al., 2010). Another powerful source of security and a sense of self-value are close relationships with others. By looking for bonds and cooperation, people understand that the world they live in might be transformed into a just and rightful place (Hart et al., 2005; Cox et al., 2008; Arrowood and Pope, 2014; Bulut, 2020; Pyszczynski et al., 2020).

### Culturally Specific Embedding of Experience in Poland: Religion and Solidarity

Terror Management Theory suggests that people’s worldviews or beliefs that help to deal with the inevitability of death are integral to the culture people live in Greenberg et al. (1986); see also: Matsumoto (2006); Valsiner (2007). Considering the cultural character of Poland, the two specific shields against terror that come to mind are religiosity and solidarity. First, 94% of Polish citizens consider themselves Catholics (Główny Urza̧d Statystyczny (GUS), 2018), and the worldview of 45% of Poles entirely coincides with the Catholic Church doctrine (Centrum Badania Opinii Społecznej [OBOS], 2018). As mentioned, religious beliefs constitute a very effective means of dealing with existential concerns (Vail et al., 2010; Wojtkowiak and Rutjens, 2011). What is crucial about Catholicism is that it conceptualizes death as a pathway toward salvation, not only symbolic but literal immortality (Greenberg et al., 1986).

What is more, Catholicism supports the view of the world where good deeds will be rewarded and where it is worth maintaining high moral standards (Bulut, 2020). As many religions do, it also brings people with similar beliefs together and creates communities whose members help and support each other (Graham and Haidt, 2010). Therefore, religiosity is connected with the second cultural-specific shield among Polish citizens: *solidarity.* In Poland, the word “solidarity” refers to social integration and mutual help between people which occur at difficult times or historically important events of nationwide significance (Kida, 2013) and is unavoidably linked semantically to the Solidarity movement (i.e., a broad antisocialist social movement that operated in the 1980s in Poland; Kida, 2013). The Solidarity movement “contributed to the process of deepening the social and professional solidarity of Polish workers, farmers and intelligentsia” (p. 22, Biela, 2020) and closely cooperated with the Catholic Church in Poland, and religious leaders openly promoted solidarity in Polish society (Biela, 2020). Religiosity and solidarity seem to constitute significant parts of Polish citizens’ worldview, and according to terror management theory, living in line with one’s worldview is likely to enhance one’s self-esteem (Pyszczynski et al., 2004). In this case, increased self-esteem may be due to faith in God’s love and being a part of a religious and broader community upon which one can count. Regardless of religious crises and the nonpracticing nature of religiosity of the young generation, people may tend to turn to God in the face of the terror related to death awareness (Arrowood and Pope, 2014).

### Managing Terror Related to the Coronavirus Disease 2019 in Polish Culture: A Hypothetical Model

In this paper, we postulate a predictive pathway model describing the process of managing the terror related to the COVID-19 pandemic through a meaningful worldview. Due to individual differences in personality disposition to worry (Meyer et al., 1990), people differ in the generality of worry over time and situations, the intensity/excessiveness of worry, and the uncontrollability of worry (Molina and Borkovec, 1994). Worry, as a natural reaction to stressful and uncertain situations, can intensify during the COVID-19 pandemic (Zysberg and Zisberg, 2020). Alternatively, individuals who concentrate on what is happening in the current moment tend not to worry (Sobol-Kwapińska et al., 2016). Concentration on the present (i.e., carpe diem) manifests in the awareness of the current moment and appreciation of its uniqueness (Sobol-Kwapińska, 2013). Such temporal orientation allows one to limit unpleasant emotions related to the past (e.g., regret, longing) and reduce worry and anxiety when thinking about the future.

We begin our theoretical model with the worry trait as a negative predictor of self-esteem and a positive predictor of the search for meaning in life (i.e., an adaptive way of stress coping). In turn, searching for meaning in life positively predicts either the expectation of solidarity due to the COVID-19 pandemic or centrality of religiosity, which also indirectly promotes belief in social values, solidary help being one of them. A worldview saturated with religious beliefs, the expectation of social solidarity, or both predicts concentration on the current moment; that is, living in the “here and now” with regards to the dictum “carpe diem” (Sobol-Kwapińska, 2013). This may be because focusing on religious experiences, feelings, and practices, as well as on helping behavior toward other people, may distract a person from concentrating on bothersome memories (e.g., frightening news stories or personal losses due to COVID-19) or pessimistic visions of future, charged with a significant probability of losing one’s life, health, loved ones, or income. Because living in line with a religious worldview should enhance positive self-regard, the centrality of religiosity should also be a positive predictor of self-esteem (i.e., another essential predictor of carpe diem). Therefore, the centrality of religiosity should also predict carpe diem indirectly (i.e., due to the mediating role of self-esteem).

## Method

### Participants and Procedure

This study was run during the COVID-19 pandemic in Poland during the time from April to May 2020. During that time in Poland, as in other European countries, people have been asked by the government to stay at home in order to prevent the further spread of COVID-19. Poles were instructed to wear face coverings and maintain a 2-m social distance while meeting people in a public space. Several restrictions have been implemented, such as closing nurseries and educational institutions (classes were run remotely) and most non-essential businesses and services.

The study used a cross-sectional design. We disseminated a battery of questionnaires online in line with the snowball sampling recruitment strategy. The sample consisted of 295 Polish citizens (68% female) ranging between 17 and 76 years old (*M* = 32.44). The sample makes it possible to identify the correlation of *r* > 0.20 between the variables at the significance level of α = 0.05 with a power of 1 – β = 0.95. Respondents did not receive payment for their participation in the study. Data was collected individually, anonymously, each person gave written consent to participate, was given all necessary information about the study, and could withdraw at any time. We controlled demographic variables to ensure that the sample was representative: half of the participants were students and more than half of the participants were employed at the time of the pandemic. One third of the subjects were single, one third were married and one third were in informal relationships. About 30 percent of the sample came from villages and small towns, about 15 percent from medium-sized cities and about 55 percent from large cities. Nearly three-quarters of the participants were Roman Catholic, and one-fifth were nonbelievers; the remaining subjects reported other Christian affiliations.

### Measures

We measured the worry trait with the 16-item Penn State Worry Questionnaire (Meyer et al., 1990). Sample items include “Many situations make me worry,” as well as “I find it easy to dismiss worrisome thoughts.” The Penn State Worry Questionnaire is unidimensional, valid measure, independent of susceptibility to social approval. It has satisfactory internal consistency in both anxiety disorder and control groups. Convergent and discriminant validity were established via correlations with measures of depression, emotional control and anxiety (Brown et al., 1992). Significantly, cognitive therapy may reduce the worry trait revealed by the Penn State Worry Questionnaire.

We investigated respondents’ search for meaning with a corresponding 5-item subscale of the Meaning in Life Questionnaire (Steger et al., 2006), which measures how much people strive to find understanding and meaning in their lives (sample item: “I am always searching for something that makes my life feel significant”). It demonstrates good convergent validity and discriminant validity from other aspects of meaning in life (e.g., it has different correlates than presence of meaning in life). Respondents assessed each item on a 7-point scale ranging from 1 (*absolutely true*) to 7 (*absolutely untrue*).

We also used the Centrality of Religiosity Scale (Huber and Huber, 2012) to probe the centrality, importance, or salience of religious meanings in respondents’ personal construct systems. It consists of 15 items assessed on a 5-point scale, ranging from 1 (*not at all*) to 5 (*a lot*). Sample items include “How often do you pray spontaneously when inspired by daily situations?” and “How important is it for you to be connected to a religious community?” Scores in Centrality of Religiosity Scale correlate positively with measures of religious identity and importance of religion for daily life and other popular measures of religiosity (Zarzycka, 2007).

We measured active and positive concentration on the present (i.e., “here and now” temporal orientation called *carpe diem*) with the Carpe Diem Scale (Sobol-Kwapińska, 2013), consisting of 10 items assessed on a 5-point Likert scale, ranging from 1 (*very untrue*) to 5 (*very true*). A sample item is “What happens in the present is very vital for my life.” The “Carpe Diem” scale demonstrates discriminant validity with other dimensions of temporal orientation and correlates with satisfaction with life.

We used three purpose-invented items to measure the expectation of solidarity due to the COVID-19 pandemic. They had one common stem (“Do you agree that the COVID-19 pandemic experience will lead to an increase in …”) but used the following different endings: “interpersonal solidarity?,” “intergenerational solidarity?,” or “empathy displayed by people toward each other?.” Respondents rated the items on a 5-point scale, ranging from 1 (*definitely disagree*) to 5 (*definitely agree*), and the items constituted one reliable factor (α = 0.78), explaining 69.4% of their variance.

Finally, we used the widely used unidimensional Rosenberg Self-Esteem Scale (Rosenberg, 1965) to probe overall feelings of self-worth (which correlate, i.e., with extraversion, emotional stability and physical health) on a 4-point scale.

## Results

Table 1 presents descriptive statistics and bivariate correlations between measured variables and demographics. Because the correlations between the variables included in the hypothetical model were as expected, we performed path analysis (with the bootstrap 95% estimation of confidence intervals, 2,000 samples) to confirm the model. The overall model fit was very good: χ^2^ (*df* = 5, *n* = 295) = 3.705, *p* = 0.593, root means square error of approximation RMSEA = 0.00 (CI90: 0.00 to 0.07), comparative fit index CFI > 0.99, and standardized root mean square residual SRMR = 0.02.

**TABLE 1:** Bivariate scale intercorrelations and descriptive statistics.

Variable	(1)	(2)	(3)	(4)	(5)	(6)
(1) Trait of worry	(0.93)	0.22***	0.06	0.04	–0.42***	–0.25***
(2) Search for meaning		(0.86)	0.18**	0.21***	–0.11	0.08
(3) Expectation of solidarity due to						
COVID-19 pandemic			(0.78)	0.25***	–0.02	0.19**
(4) Centrality of religiosity				(0.96)	0.13*	0.22***
(5) Self-esteem					(0.88)	0.31***
(6) Carpe diem						(0.80)
*M*	3.16	4.99	8.07	2.86	2.98	3.76
*SD*	0.84	1.26	2.29	1.21	0.59	0.58
*Min*	1.19	1.00	3.00	1.00	1.00	2.10
*Max*	4.98	7.00	13.00	5.20	4.00	5.00

*Correlation coefficients: Pearson’s r for all variables. Scale internal reliabilities (Cronbach’s α) in parentheses on diagonal. N = 295.*

* p < 0.05, **p < 0.01, ***p < 0.001.

**FIGURE 1:**
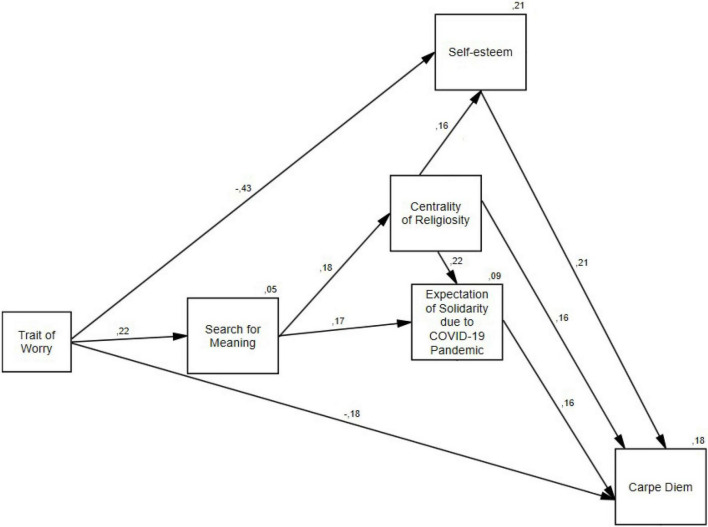
Path model: search for meaning, centrality of religiosity, expectation of solidarity, and self-esteem and carpe diem orientation. *N* = 295.

The empirical model is presented in Figure 1. The worry trait was a negative predictor of self-esteem; that is direct (i.e., total) effect of the worry trait on self-esteem was β = –0.42 (CI95: –0.53 to –0.32). Indirect, direct, and total effects of the worry trait on carpe diem were β = -0.08 (CI95: –0.14 to –0.02), β = –0.18 (CI95: –0.30 to –0.05) and β = –0.26 (CI95: –0.37 to –0.14), respectively. However, due to the positive predictive effect of the worry trait on the search for meaning [direct, i.e., total effect was β = 0.22 (CI95: 0.10 to 0.34)], the worry trait indirectly predicted paths leading to carpe diem through three positive predictors of the latter; namely, (a) centrality of religiosity, (b) expectation of solidarity due to COVID-19 pandemic, and (c) self-esteem.

The positive indirect (i.e., total) effect (i.e., mediated by search for meaning) of the worry trait on centrality of religiosity was β = 0.04 (CI95: 0.01 to 0.08]. In turn, the positive direct (i.e., total) effect of search for meaning on centrality of religiosity was β = 0.18 (CI95: 0.06 to 0.29), and the positive direct (i.e., total) effect of centrality of religiosity on expectation of solidarity was β = 0.22 (CI95: 0.10 to 0.33). Centrality of religiosity was also a positive predictor of self-esteem (direct, i.e., total effect equal to β = 0.16 (CI95: 0.04 to 0.28). In addition, indirect (i.e., mediated by centrality of religiosity), direct, and total effects of search for meaning on expectation of solidarity were β = 0.04 (CI95: 0.01 to 0.07), β = 0.17 (CI95: 0.06 to 0.27), and β = 0.21 (CI95: 0.09 to 0.31), respectively. In general, indirect (i.e., total) effect of search for meaning on carpe diem was β = 0.07 (CI95: 0.03 to 0.11).

Most importantly, indirect (i.e., mediated by expectation of solidarity and self-esteem), direct, and total effects of centrality of religiosity on carpe diem were as follows: β = 0.07 (CI95: 0.03 to 0.12), β = 0.16 (CI95: 0.04 to 0.28), and β = 0.23 (CI95: 0.12 to 0.33). Furthermore, the direct (i.e., total) effect of expecting solidarity on carpe diem was β = 0.16 (CI95: 0.05 to 0.27), and the direct (i.e., total) effect of self-esteem on carpe diem was β = 0.21 (CI95: 0.05 to 0.27). In sum, the model predicted 18% of variance of carpe diem, illustrating a terror management strategy during the COVID-19 pandemic by means of worldview (religiosity and expectation of solidarity) and self-esteem.

## Discussion

In this study, we tested the model proposed by the TMT, that predicts people coping behaviors in the life-threatening situations, what can be applied to the case of COVID-19 pandemic. According to TMT, people try to protect themselves from experiencing anxiety by holding onto particular worldviews, that help them maintain their self-esteem and, as a result, manage challenging situations. In our study we examined worldviews that are culturally specific for Polish citizens, that is religiosity and solidarity. To asses Poles’ emotional condition we examined their tendency to worry about the future – which is likely to arise due to pandemic – together with a concentration on the current moment (“carpe diem” orientation), which helps reduce worry and anxiety. We investigated the mediating role of the centrality of religiosity, the expectation of solidarity due to the COVID-19 pandemic, and self-esteem in the relationship between the worry trait and carpe diem temporal orientation (Sobol-Kwapińska, 2013).

We confirmed the postulated model, advocating the roles of worldview and self-esteem in terror management (Solomon et al., 1991). According to the results, the model explains 18% of the carpe diem orientation, and it may be interpreted as follows. Although directly and negatively related to self-esteem and a “here and now” temporal perspective, the worry trait may also predict the search for meaning in life (i.e., activity that in turn activates one’s focus on the current moment). This is because the search for meaning predicts both a turn to religiosity and the expectation of interpersonal solidarity (i.e., two perspectives that make up the worldview of Polish citizens). Living in accordance with a religious worldview allows one to predict higher self-esteem. Moreover, self-esteem, the centrality of religiosity, and the expectation of solidarity are positive predictors of a carpe diem temporal orientation that theoretically protects against worry and might be a religiosity-induced adaptive way of being “here and now” during the COVID-19 pandemic (cf. Kranz et al., 2020).

Our results reveal that reliance on religion and solidarity in the Covid-19 crisis is a theme present in the collective memory of the Polish nation, strengthening their in-group identification. We, therefore, show that such traits as religiosity and solidarity form the cultural worldview that serves as a boost to self-esteem, allowing one to defend against terror and anxiety. More particularly, the need for solidarity coupled with religiosity leads to the view that all people – other people and oneself – are valuable, forming an important source of self-esteem. Moreover, our studies show that the ways of increasing one’s self-esteem and strengthening the ability to adapt to the here-and-now situation are importantly embedded in one’s cultural context (compare: Chakkarath, 2012; Romano et al., 2021).

The results of our study are consistent with previous research on the positive role of religiosity in dealing with uncertainty, that without a doubt applies to the unexpected and unstable situation of pandemic (Bardeen and Michel, 2017; Howell et al., 2019). Religiosity has been identified as a factor that fosters adaptive ways of emotion regulation (Vishkin et al., 2019), and predicts emotional life satisfaction and mental health (Platsidou, 2012). Recently it has been observed that the COVID-19 pandemic impacted the people’s tendency to seek comfort and solace in religion (Bentzen, 2020; see also Rigoli, 2021). For example, it has been reported that the Google searches for prayers in 95 countries across the globe have risen up significantly at the onset of pandemic and amounted to the highest number recorded in March 2020 (Bentzen, 2020). Prayers searches applied to all major religions, which allows to consider it as a global phenomenon. At the same time is was observed that the searches for prayers rose more in more religious countries, where religion may play important role in coping with anxiety and emotional distress (ibidem) – as in Poland (see also Szałachowski and Tuszyńska-Bogucka, 2021).

There is also a growing body of research on the significance of social solidarity in the times of pandemic (Elcheroth and Drury, 2020; Igwe et al., 2020; Mishra and Rath, 2020). It is highlighted that people seek for the sense of community, as it helps them to deal with existential threats (Sani et al., 2009). As we mentioned, in Poland, solidarity is understood not only as the sense of community or connection with other members of society, but it also as an imperative to take care of others, especially those in need (Kida, 2013). The fact that Polish society has experienced the power of coming together to help each other in the critical situations with the uprising of the “Solidarity” movement, may be the reason why Poles expect social solidarity in the case of COVID-19 pandemic. It is coherent with the studies that show that communities’ understanding of their history, collective memories and meaningful narratives motivate them to deal with the situation of pandemic (Elcheroth and Drury, 2020).

Significant limitations of this study include correlational design based on declarative questionnaires and the resulting path model, which does not allow conclusions about cause-and-effect relationships. The emerging question is whether our correlational model of terror management during the COVID-19 pandemic may lay the foundations for future experimental research on the potential changes in relatively stable dimensions of personality (i.e., tendency to worry, centrality of religiosity, and carpe diem). Expecting short-term changes in patterns of thoughts, feelings, and behaviors rather than long-lasting dispositional changes in personality due to the COVID-19 pandemic seems more likely (cf. Sutin et al., 2020). However, one should be vigilant because living during a pandemic is somewhat reminiscent of living in a war zone, and the latter has already proved to change the personalities of its survivors (Murthy and Lakshminarayana, 2006).

## Data Availability Statement

The raw data supporting the conclusions of this article will be made available by the authors, without undue reservation.

## Author Contributions

ŁM: manuscript writing, theoretical background, study design, data analysis, and discussion of the findings. AG: study design, data collection, theoretical background, and discussion of the findings. OG: data analysis and discussion of the findings. PG: theoretical background and discussion of the findings. AK: discussion of the findings and manuscript writing. All authors contributed to the article and approved the submitted version.

## Conflict of Interest

The authors declare that the research was conducted in the absence of any commercial or financial relationships that could be construed as a potential conflict of interest.

## Publisher’s Note

All claims expressed in this article are solely those of the authors and do not necessarily represent those of their affiliated organizations, or those of the publisher, the editors and the reviewers. Any product that may be evaluated in this article, or claim that may be made by its manufacturer, is not guaranteed or endorsed by the publisher.
